# High Glucose Alters Extracellular Vesicle Protein Composition and Promotes EV-Mediated Monocyte Adhesion to Endothelial Cells

**DOI:** 10.3390/ijms27188107

**Published:** 2026-09-11

**Authors:** Verônica Vitória Vedam, Gisele Tatiane Soares da Veiga, Líndice Mitie Nisimura, Letusa Albrecht

**Affiliations:** 1 Fundação Oswaldo Cruz, Fiocruz Paraná, Instituto Carlos Chagas, Laboratório de Pesquisa em Apicomplexa, Curitiba 81350-010, PR, Brazil; vvedam@aluno.fiocruz.br (V.V.V.); gisele.veiga@fiocruz.br (G.T.S.d.V.); lindice.nisimura@fiocruz.br (L.M.N.); 2 Fundação Oswaldo Cruz, Fiocruz Paraná, Instituto Carlos Chagas, Laboratório de Imunologia Molecular, Celular e Inteligência Artificial, Curitiba 81350-010, PR, Brazil

**Keywords:** hyperglycemia, inflammation, proteomics, metabolism, innate immunity, endothelial dysfunction

## Abstract

Diabetes mellitus prevalence is rising globally, linked to persistent hyperglycemia and endothelial dysfunction. Extracellular vesicles (EVs) play a role in diabetes pathology, involving complex intercellular communication that influences endothelial response. However, the mechanisms by which EVs contribute to endothelial dysfunction under hyperglycemia remain unknown. To investigate the effect of glucose-altered EVs on endothelial cells, we isolated EVs from HBMECs and THP-1 cells under normal- and high-glucose conditions (5.5 and 33 mM). We used NTA, electron microscopy, and LC-MS/MS for EV characterization. HBMECs in RPMI’s default glucose (11 mM) were exposed to 100 ng/mL of each EV condition, and adherent THP-1 CFSE+ cells were quantified. Long-term high glucose activated HBMECs without altering cell viability. Glucose level altered EV secretion, content, and function. For HBMECs, high glucose decreased EV release but shifted their cargo toward metabolism, barrier disruption, and neuronal protein content. For THP-1 cells, high glucose kept the EV release rate and shifted the cargo toward an inflammatory activation profile. Functionally, high-glucose EVs (mainly THP-1-derived) showed a pro-adhesive effect on endothelial cells. Here, we describe the impact of glucose on EV biology and how glucose-altered EVs can influence inflammation and endothelial responses, highlighting the importance of glycemic control in diabetic patients.

## 1. Introduction

Diabetes mellitus (DM) is a chronic metabolic disorder with a rising global prevalence. It is associated with persistent hyperglycemia, resulting from an insulin production deficiency (type 1) or insulin resistance (type 2). In addition to disrupting glycemic homeostasis, DM can lead to a range of micro- and macrovascular complications, including atherosclerosis, hypertension, and peripheral arterial disease [1,2,3,4]. Recently, a higher risk of cognitive dysfunction has been linked to hyperglycemia and increased blood–brain barrier (BBB) permeability [5,6]. Therefore, diabetes and its vascular complications are associated with high morbidity and can severely impair both health and quality of life [7].

Vascular complications are often attributed to alterations in endothelial cells, which are crucial for maintaining the integrity and function of the blood vessel lining [8,9,10]. Beyond its physical role as a barrier, the endothelium functions as a highly specialized and dynamic organ, crucial for physiological processes and maintaining homeostasis [10]. Under normal conditions, endothelial cells actively regulate the inflammatory response by maintaining a dynamic balance that prevents excessive adhesion of leukocytes and other inflammatory cells to the vascular wall, with a basal production of nitric oxide (NO), for example [11,12,13].

Prolonged exposure to elevated blood glucose levels can lead to chronic inflammation and oxidative stress, exacerbating endothelial dysfunction [8,14,15]. This process results in vascular leakage and abnormal permeability [16,17]. In the DM context, persistent hyperglycemia can trigger changes in the expression of adhesion molecules and pro-inflammatory factors, creating an environment favorable to adhesion and infiltration of inflammatory cells [11,18,19,20,21].

The participation of extracellular vesicles (EVs) in inflammation has generated significant interest in DM and its vascular complications [22,23,24,25,26]. EVs are small membrane-bound structures released by cells that have proteins, lipids, and RNAs. They are crucial mediators in cell communication and have been associated with various pathological processes [27]. In a hyperglycemic environment, altered EVs may contribute to endothelial dysfunction by promoting inflammation and modifying endothelial responses [28,29,30]. This can further exacerbate vascular damage and increase the risk of complications associated with diabetes, such as dementia and other cognitive dysfunctions [3,5,9].

However, the precise mechanisms by which EVs contribute to endothelial dysfunction are not yet fully understood. The specific effect of hyperglycemia on EV release from immune cells and the way these EVs, in turn, affect endothelial function remains unknown. In this study, we characterized EVs derived from human cells (HBMEC and THP-1 lines) subjected to prolonged exposure to high- and normal-glucose concentrations; we evaluated their proteome and functional effect on monocyte adhesion to the endothelium, aiming to provide insights into the role of these EVs in endothelial dysfunction, which is frequently observed in diabetic patients and individuals with metabolic disorders.

## 2. Results

### 2.1. High Glucose Increases HBMEC Expression of Endothelial Activation Markers

We evaluated human brain microvascular endothelial cell (HBMEC) cultures under continuous exposure to high- and normal-glucose concentrations. To simulate human physiological and pathological conditions, the HBMEC monolayer was kept in normal (5.5 mM, equivalent to 99.9 mg/dL) and high-glucose (33 mM, equivalent to 594 mg/dL) media for at least two cell passages before evaluation, representing long-term exposure.

**Figure 1:**
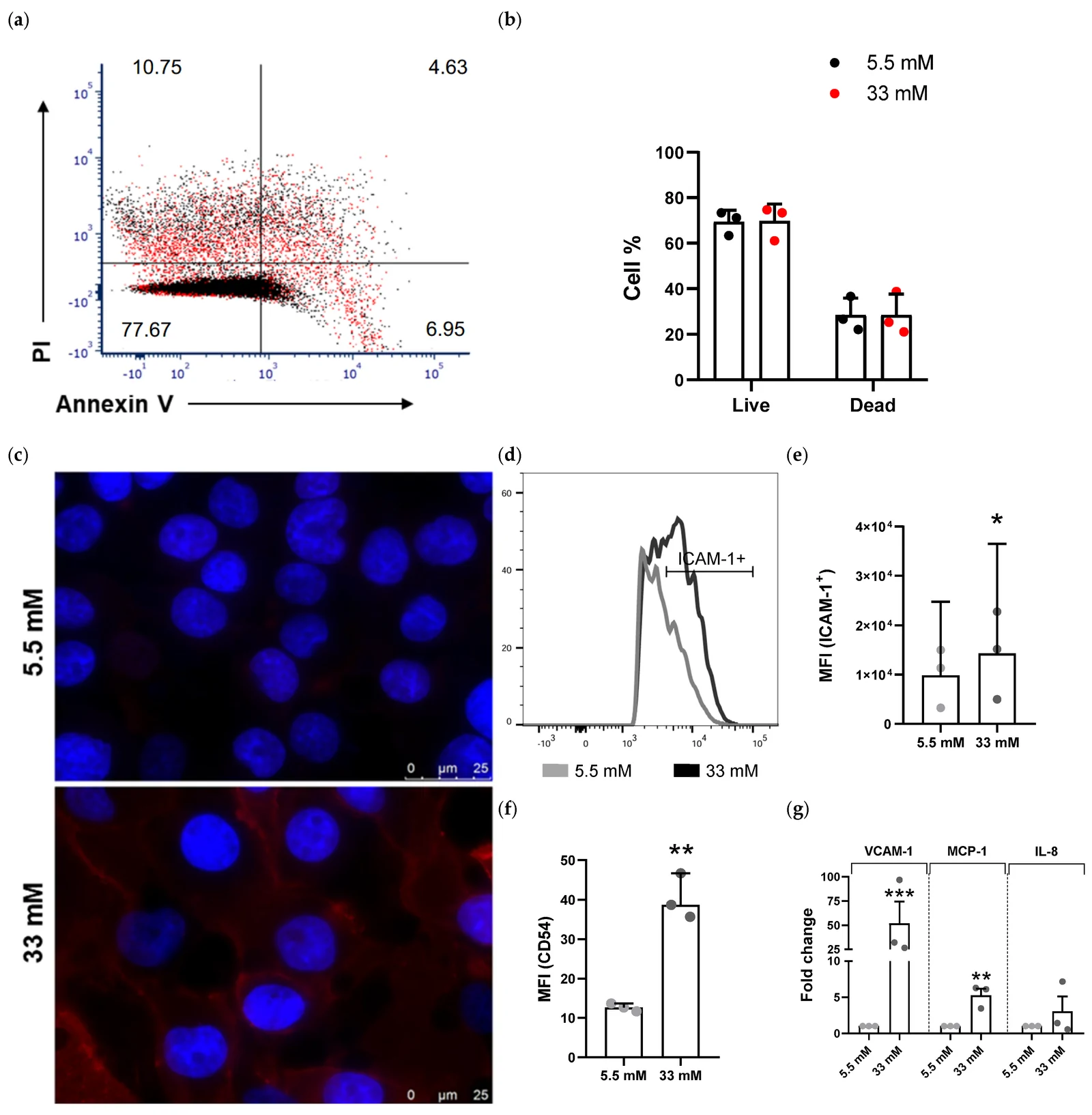
High glucose induces human endothelial activation in vitro. (**a**) Cell viability of HBMECs cultured in 5.5 mM (black dots) and 33 mM (red dots) glucose, labeled with Alexa Fluor 488 Annexin V and propidium iodide (PI), evaluated by flow cytometry. The values shown in each quadrant represent cell percentages after software normalization relative to the mode. (**b**) Live/dead ratio from three independent cell viability experiments. Bars represent the mean and standard deviation (SD) of biological replicates. (**c**) Representative immunofluorescence and (**d**) flow cytometry analysis of intercellular adhesion molecule 1 (ICAM-1) in HBMEC cultured in 5.5 mM and 33 mM glucose. (**e**) Median fluorescence intensity (MFI) of the ICAM-1+ gate from three independent experiments/biological replicates (* *p* < 0.05) and (**f**) MFI of the red fluorescence channel (Alexa Fluor 546) from three technical replicates (** *p* < 0.001). Bars represent the median and median absolute deviation (MAD) of the replicates. (**g**) Relative mRNA levels of HBMECs cultured in 5.5 mM and 33 mM glucose for VCAM-1, MCP-1, and IL8 genes by qPCR. The 2^−ΔΔCT^ method was used; fold change relative to *β-actin*. Student’s *t*-test *** *p* < 0.0001 ** *p* < 0.001. Data from three independent experiments; bars stand for the mean and standard error (SEM) of three biological replicates.

No difference in cell viability was seen between HBMECs cultured in 5.5 mM and 33 mM glucose (Figure 1a,b), nor was there labeling of specific apoptotic (Annexin-V+/PI−) and necrotic (Annexin-V−/PI+) processes. More than 75% of cells remained viable after 48 h of culture (Figure 1a,b). Given the sensitivity of endothelial lineages to manipulation, the total percentage of dead cells (apoptosis/necrosis) was largely attributable to cell processing (Figure 1b).

The expression of intercellular adhesion molecule 1 (ICAM-1), also known as CD54, increased in endothelial cells kept under high-glucose conditions (Figure 1c,f). Flow cytometry analysis revealed a nearly 1.5-fold increase in median fluorescence intensity (MFI) at 33 mM glucose (Figure 1d,e).

Other markers of endothelial activation showed changes in messenger RNA (mRNA) levels in HBMECs under high-glucose conditions: vascular cell adhesion molecule 1 (VCAM-1) showed a 51.8-fold increase, and monocyte chemoattractant protein 1 (MCP-1) showed a 5.3-fold increase (Figure 1g). Interleukin 8 (IL-8) increased threefold between glucose conditions, but the change was not significant.

### 2.2. EV Secretion Varies Depending on the Glucose Level of the Progenitor Cell Type

**Figure 2:**
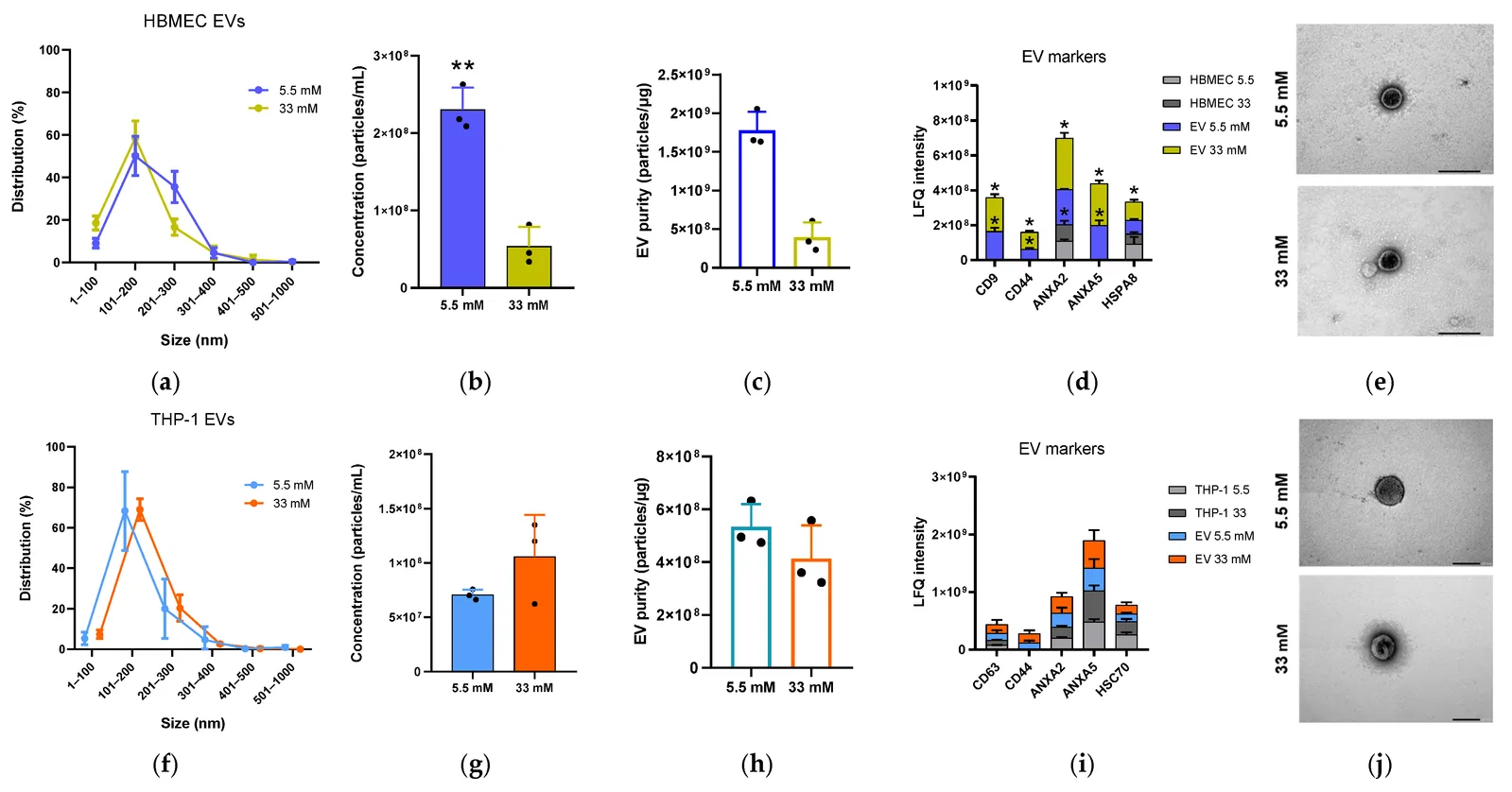
Profile of HBMEC and THP-1 extracellular vesicles (EVs) at different glucose levels. (**a**,**f**) NTA for size distribution, (**b**,**g**) particle concentration, and (**c**,**h**) the estimated purity of EVs from 5.5 mM and 33 mM glucose cultures. Bars represent the mean and SD of three biological replicates; unpaired *t*-test (** *p* < 0.001). (**d**,**i**) Raw intensities of EV markers CD9, CD63, CD44, ANXA2, ANXA5, HSPA8, and HSC70 from HBMEC and THP-1 cell extracts and their EVs. LFQ: label-free quantification. Bars represent the mean and SD of three biological replicates from each group; multiple *t*-tests (* *p* < 0.05). (**e**,**j**) Representative EV morphology by TEM, detail of the lipid bilayer. Scale bar: 100 nm. All data shown are from three independent EV batch isolations.

To characterize the EV release under high- and normal-glucose conditions, we quantified isolated EVs from HBMEC and THP-1 by NTA. The glucose level did not alter the EVs’ size distribution. Both cell lines showed homogeneous EV populations with diameters of 100–300 nm (Figure 2a,f). EVs derived from the same number of cells showed differences in concentration depending on the glucose condition (Figure 2b,g).

For HBMEC, the release of EVs decreased in high-glucose medium (Figure 2b)—about four times fewer particles/mL. For THP-1, the release showed no statistical difference (Figure 2g). The particle-to-protein ratio ranged from 2 × 10^8^ to 2 × 10^9^ particles/µg protein (Figure 2c,h). This suggests that the samples have low non-vesicular protein contamination.

The EVs’ morphology, obtained by transmission electron microscopy (TEM), confirmed the presence of particles consistent with EVs in all samples (Figure 2e,j). The size distribution was consistent with the NTA, with a visible diameter greater than 100 nm.

The proteomic-based analysis showed the presence of putative extracellular vesicle markers, namely, CD9, CD44, annexins (ANXA2 and ANXA5), and heat shock protein family (HSPA8), which were enriched in EV samples from HBMEC compared to the lysate of their cell source (Figure 2d). This enhancement occurred despite the glucose concentration. For THP-1 EVs, we also identified classic EV markers: CD63, CD44, ANXA2, ANXA5, and HSC70 (Figure 2i). However, the LFQ intensity values did not differ from those of the respective cell lysate in this case.

### 2.3. Glucose-Rich Environment Alters EVs’ Protein Composition

**Figure 3:**
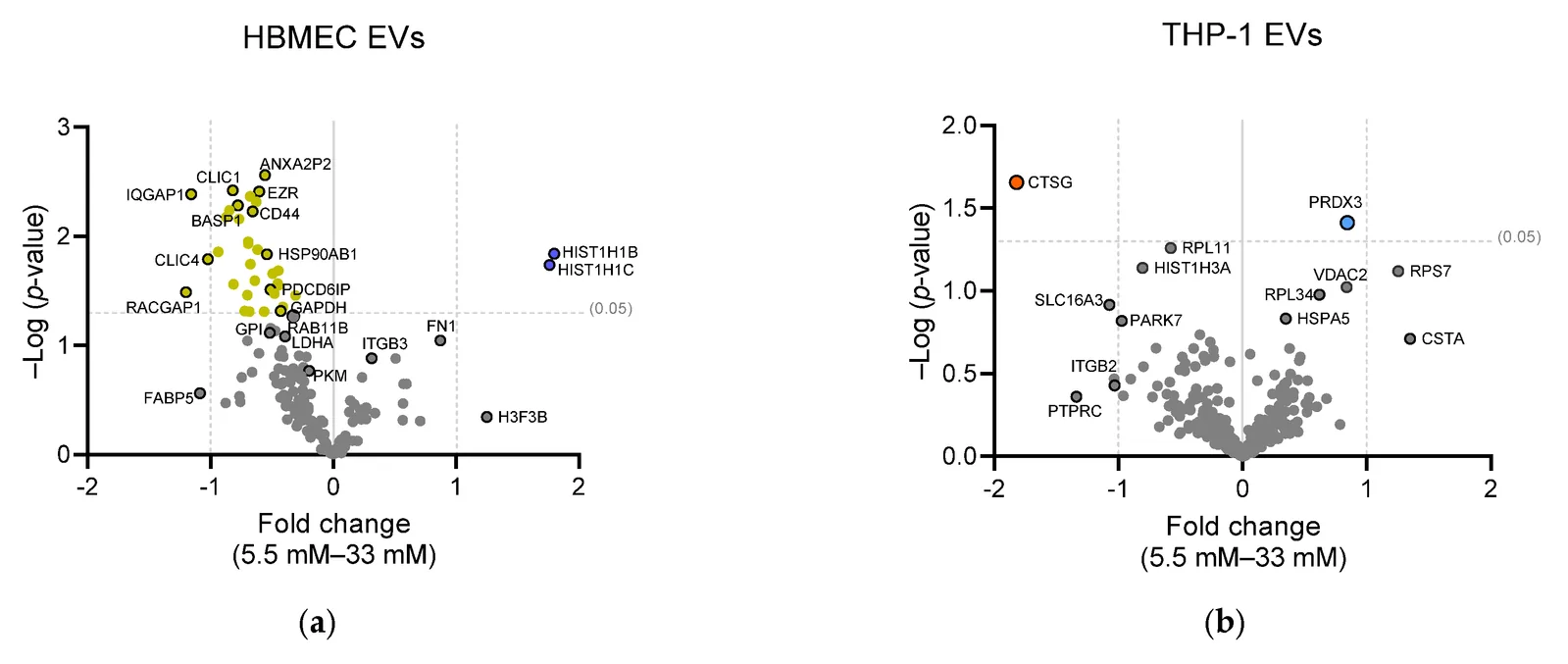

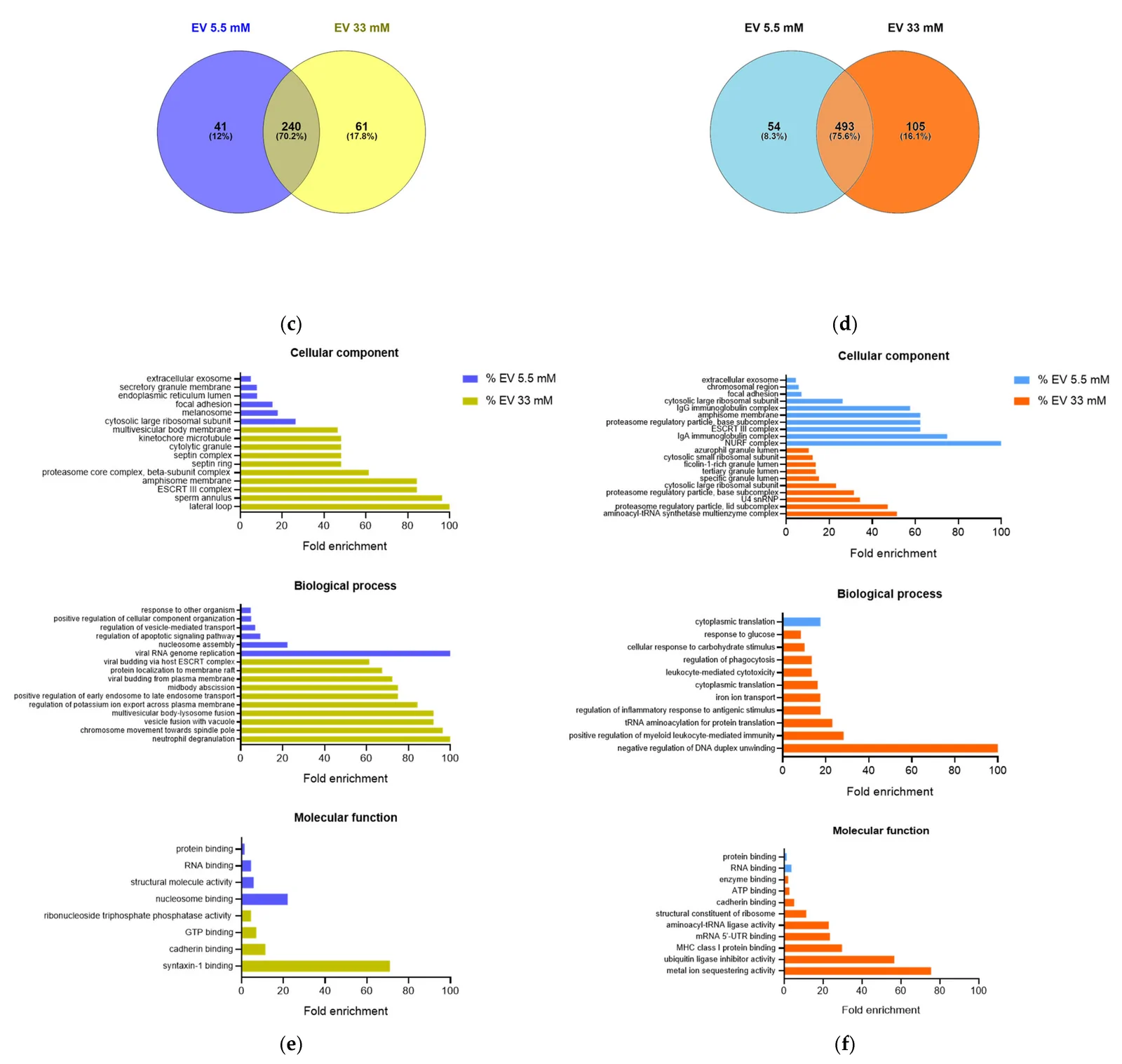
Proteome of HBMEC and THP-1 extracellular vesicles (EVs) in normal- and high-glucose medium. (**a**,**b**) Differences in protein expression between normal (5.5 mM) and high (33 mM) glucose-isolated EVs. Colored markings represent *p* < 0.05, gold/orange for 33 mM and blue for 5.5 mM. (**c**,**d**) Venn diagrams of identified proteins as common or not between glucose exposures in the EVs (three biological replicates). (**e**,**f**) Gene ontology enrichments for the 102 non-shared proteins (HBMEC) and the 159 non-shared proteins (THP-1) identified in EVs derived from 5.5 mM and 33 mM glucose cultures. PANTHER classification system was used to analyze complete cellular components, biological processes, molecular functions, and pathways. Significant GO terms are shown up to the 10 most overrepresented terms.

The total protein content of EVs was assessed by mass spectrometry (LC-MS/MS). Having established at least two unique peptides in two replicates for presence, we identified 342 distinct proteins in HBMEC EVs. Of these, 240 proteins were common to both glucose conditions, and 102 proteins differed between the 5.5 mM and 33 mM glucose conditions (Figure 3c, Table S1). Based on LFQ intensity values from all three biological replicates, 37 proteins were found to be differentially expressed (*p* < 0.05) in 33 mM EVs, among them: BASP1, CLIC1 and CLIC4, GAPDH, heat shock protein 90 family (HSP90AB1), and the CD44 marker (Figure 3a). In 5.5 mM HBMEC EVs, two proteins were found to be differentially expressed (*p* < 0.05): two linker histone (H1) isoforms.

The gene ontology (GO) analysis of the 102 non-shared proteins identified in each glucose condition showed GO term enrichment across all three aspects, namely, cellular component, biological process, and molecular function, especially in 33 mM glucose HBMEC EVs (Figure 3e). These included the ESCRT-III complex and other endosomal membrane-related terms, as well as the enrichment of cytokinesis components and processes, and syntaxin-1 binding function.

In 5.5 mM glucose HBMEC EVs, the enriched terms were related to regulation, response to other organisms, focal adhesion, and extracellular exosome class (Figure 3e). Altogether, these results were similar to the proteome profile obtained from the respective progenitor cell lysate (Supplementary Materials), reinforcing the influence of the specific cell state and/or its surroundings on EV composition and consequently their function.

In EVs from THP-1 monocytes, we identified 652 distinct proteins using the same strategy described above. Of these, 493 proteins were common to both glucose conditions, and 159 proteins differed between the 5.5 mM and 33 mM glucose conditions (Figure 3d, Table S2). Despite the greater number of identifications, only two proteins were differentially expressed (*p* < 0.05) between 5.5 mM and 33 mM EVs (Figure 3b). The proteins were cathepsin G (CTSG) in 33 mM THP-1 EVs and peroxiredoxin 3 (PRDX3) in 5.5 mM THP-1 EVs.

GO analysis of the 159 proteins identified as non-shared between glucose conditions revealed greater term enrichment in high-glucose THP-1 EVs (Figure 3f). THP-1 33 mM EVs showed terms related to secretion, glucose response, inflammation, cytotoxicity, and antigen presentation (MHC-I). For THP-1 5.5 mM EVs, terms related to IgA and IgG immunoglobulin complexes and focal adhesion were found. The PANTHER pathways overrepresentation test showed no statistically significant results for any analyzed condition. 

### 2.4. THP-1 EVs Promote Adhesion in Human Brain Endothelial Cells

**Figure 4:**
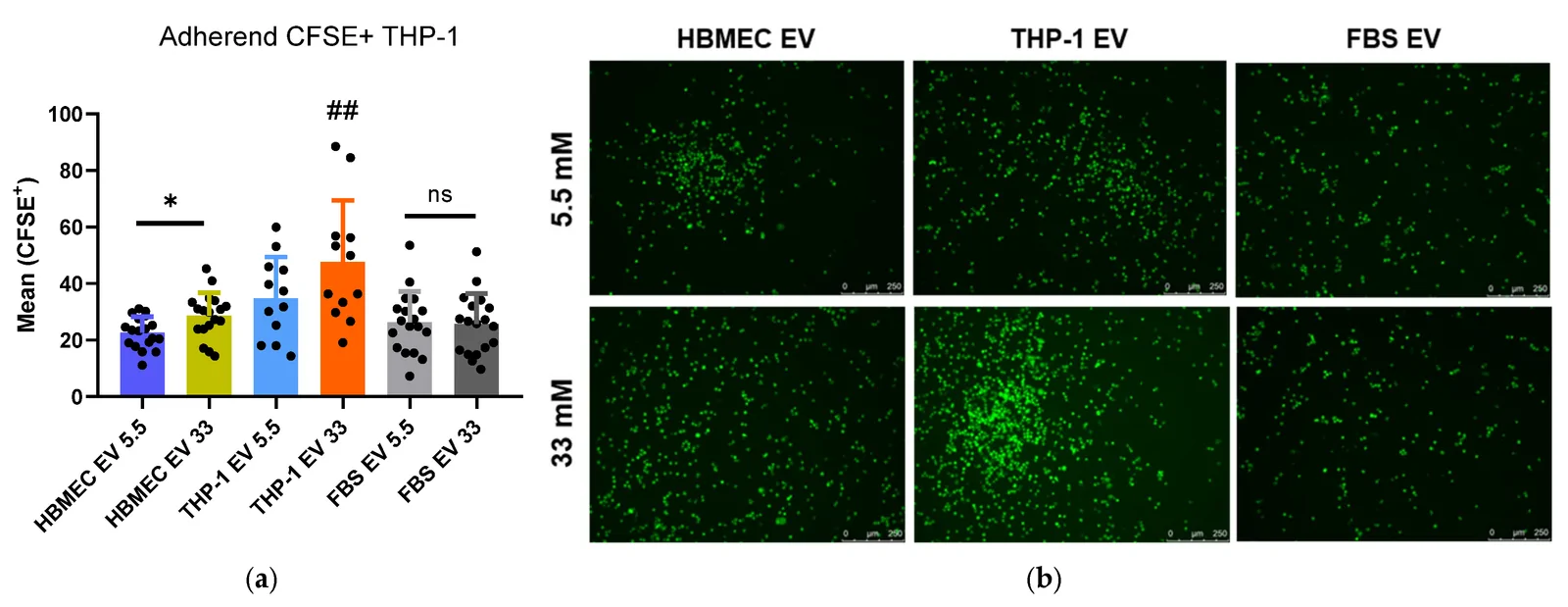
Increased monocyte adhesion to endothelial monolayer after EV stimulation from high-glucose cells. (**a**) HBMEC monolayer cultured at 11 mM glucose was treated for 16 h with EVs (100 ng/mL) isolated from HBMEC and THP-1 cells at 5.5 mM and 33 mM glucose or received just RPMI media + 10% FBS (non-EV-depleted) with 5.5 mM and 33 mM glucose. About 1.2 × 10^5^ THP-1 cells labeled with CFSE were added for 1 h. After four washes, the adherent CFSE+ cells were imaged and quantitatively analyzed using ImageJ 1.54g version. Bars represent mean fluorescence intensity with SD, from three independent experiments (black points are different image fields). Unpaired *t*-test: * *p* < 0.05 or ns: not significant. Ordinary one-way ANOVA: ^##^ *p* < 0.001. (**b**) Representative images of each condition. Bars: 250 µm.

**Figure A1:**
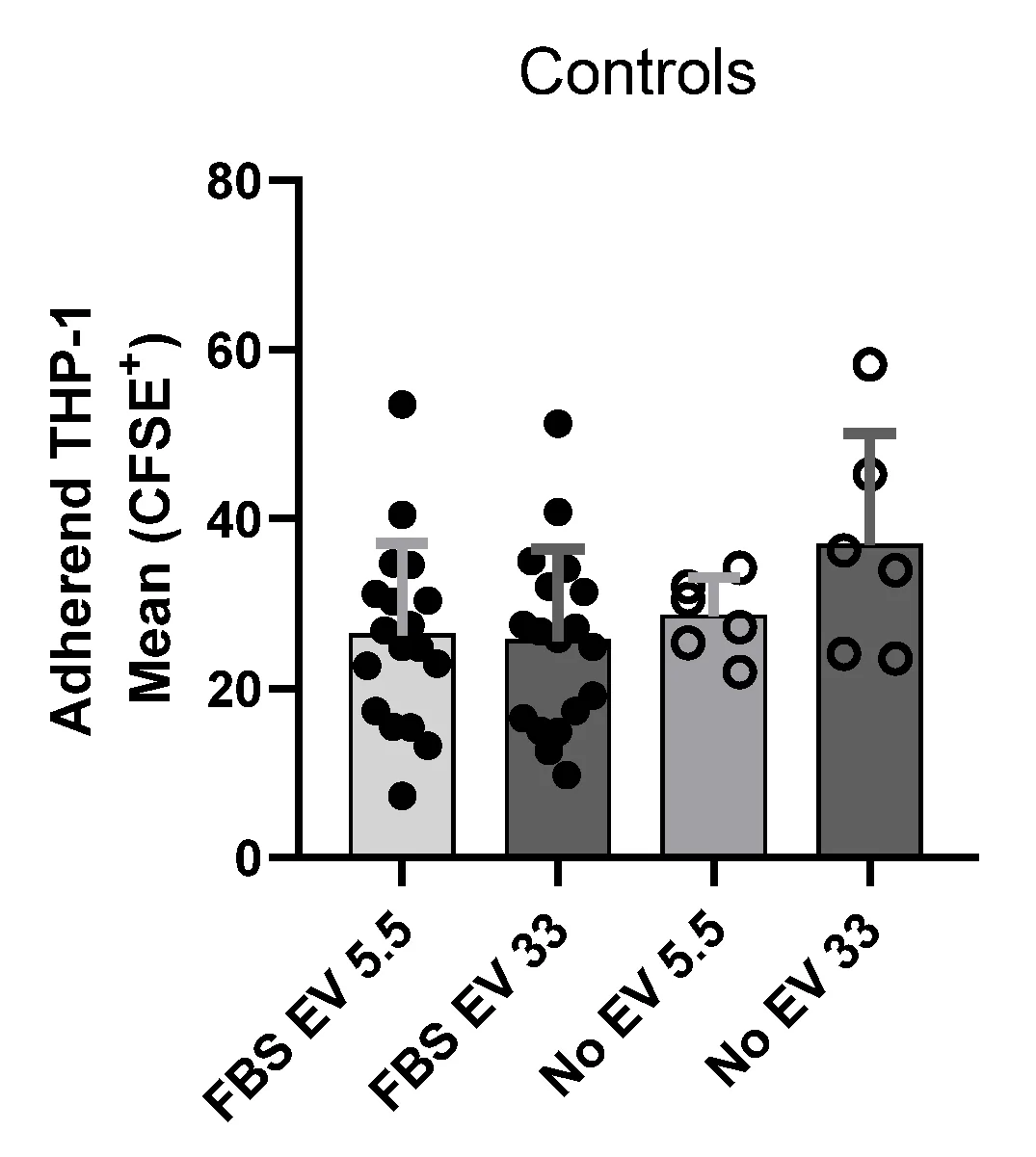
Unrelated and absence of extracellular vesicles (EVs) in cell adhesion assay. HBMEC monolayer cultured at 11 mM glucose received RPMI with 10% FBS (FBS EV condition) or 10% FBS EV-free (No EV condition) with 5.5 mM and 33 mM glucose. About 1.2 × 10^5^ THP-1 cells labeled with CFSE were added for 1 h. After four washes, the adherent CFSE+ cells were imaged and quantitatively analyzed using ImageJ. Bars represent mean fluorescence intensity with SD from three independent experiments (black points are different image fields). Unpaired *t*-test was not significant (*p* > 0.05).

To assess whether EVs derived from immunovascular cells exposed to high- and normal-glucose conditions could mediate endothelial adhesion, we performed an assay using an HBMEC monolayer at 11 mM glucose (RPMI’s default), representing a neutral/intermediate condition. We used the EV protein dosage for normalization (set as 100 ng/mL). From this design, we observed increased monocyte adhesion from high-glucose EV stimuli, especially from 33 mM THP-1 EVs (Figure 4). This increase was seen compared with the corresponding 5.5 mM EV stimulus, as well as to unrelated EVs (FBS-derived EVs) and the absence of EVs (Figure A1).

The relative enhancement of CFSE+ THP-1 adherence was greater after EV treatment with a high-glucose EV background. HBMEC 33 mM EVs increased cell adhesion compared with HBMEC 5.5 mM EVs (Figure 4a). The condition that most enhanced adherence was 33 mM THP-1 EVs. Media supplemented with 10% standard FBS (FBS EV: unrelated EV condition) or 10% EV-depleted FBS (No EV condition) showed no difference among glucose concentrations (Figure A1). This indicates that EVs themselves take part in the leukocyte adhesion process, not the environmental glucose level alone. It also suggests that EV-altered protein composition under high glucose can be converted into EV function.

In summary, our results suggest that a glucose-rich environment affects not only the endothelial cell itself, raising its activation markers, but also its EV secretion capacity. For the HBMEC cell line, high glucose decreased EV release. Our proteomic data corroborate that EV composition corresponds to the metabolic and/or inflammatory state of the progenitor cell. Furthermore, isolated EVs from THP-1 can promote increased monocyte adhesion in vitro.

## 3. Discussion

DM is associated with endothelial and monocyte activation. Hyperglycemia is one of the factors responsible for this activation [19,21]. Also, altered extracellular vesicles (EVs) from innate immune cells can contribute to the development of DM and diabetic complications [9,30,31,32].

Our study supports that human endothelial cells (HBMEC lineage) are activated by elevated glucose levels (33 mM). This was shown by the expression of the well-known endothelial activation markers: ICAM-1, VCAM-1, and MCP-1. Since our experimental conditions consisted of long-term exposure to high glucose (two or more cell passages), without an osmotic control, we analyzed cell death by apoptosis (annexin V) and necrosis (PI). There was no meaningful change in cell death percentages between normal- and high-glucose HBMEC cultures, which may indicate the plasticity/resistance of brain endothelial cells.

To investigate whether EVs in hyperglycemia mediate endothelial dysfunction and promote inflammation, we isolated and characterized EVs from HBMEC and THP-1 cultures under 5.5 mM and 33 mM glucose conditions. We did not separate EV subtypes; following ISEV/MISEV2023 recommendations, we utilize the term “extracellular vesicles” throughout this work [33].

Our proteomic characterization identified EV hallmarks of transmembrane (CD9, CD63, CD81, CD47, HLA-A) and cytosolic proteins (HSC70/HSPA8 and annexins) in all of our EV samples. In total, 25–30% of the identified HBMEC and THP-1 EV proteome was distinct between 5.5 mM and 33 mM cultures.

Data from the literature commonly show an increase in EV secretion in the context of diabetes/hyperglycemia [34,35,36]. This has been demonstrated in vivo with EVs isolated from the blood and/or urine of humans and mice, and in vitro with ex vivo tissues and other endothelial cell lines (such as BMVEC and HUVEC). However, our EV isolation protocol (24 h incubation with FBS EV-depleted) consistently revealed a decrease in HBMEC EV concentration under high-glucose exposure. THP-1 showed no significant difference in EV concentration between glucose conditions, using the same protocol.

**Figure A2:**
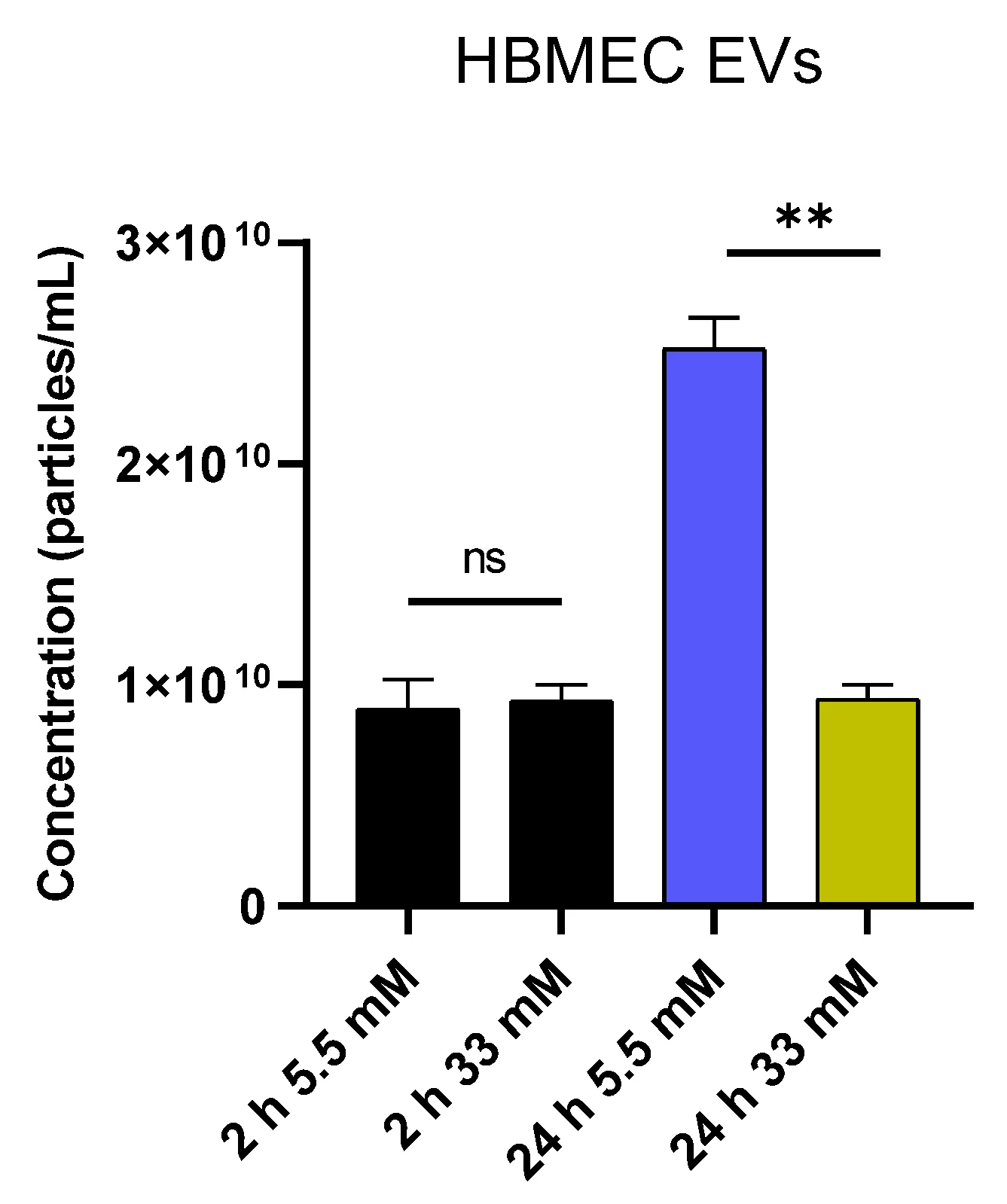
HBMEC EV isolation after 2 h or 24 h of FBS EV-free incubation. NTA particle concentration of EVs from HBMECs cultured with 5.5 mM or 33 mM glucose in RPMI + 10% FBS-free for 2 h and 24 h. Bars represent the mean and SD; unpaired *t*-test: not significant (ns) and ** *p* < 0.001.

Our group has shown that HBMEC-line cultivated with FBS EV-depleted for distinct incubation periods (2 h and 24 h) produces EVs with different physical characteristics and protein content over time [37]. NTA showed no difference in HBMEC EV concentration between 5.5 mM and 33 mM glucose during 2 h incubation (Figure A2). The 24 h incubation was standardized because 2 h EVs exhibited a cellular stress and inflammation profile compared to 24 h EVs [37].

Therefore, we argue that the applied protocol does not mask possible high-glucose EV hypersecretion and that the HBMEC line truly drops EV release in long-term exposure to 33 mM glucose. Considering that most studies involve short-term (24–30 h) exposure of endothelial cells to similar glucose concentrations (25 mM) [35,36]. The literature describing hyperglycemia-induced increases in EV secretion may be reporting an acute effect.

The implication of this finding in diabetic individuals, of a relevant reduction in brain endothelium EV release, could signify a microenvironment restriction of intercellular communication. This would potentially contribute to endothelial dysfunction and the compromise of BBB integrity under chronic hyperglycemia [5,6].

Beyond changes in HBMEC EV secretion, glucose concentration has also altered the protein EV profile. Taking 5.5 mM glucose as baseline, EVs showed high expression of histones (H1.5, H1.2, and H3.3), consistent with gene ontology (GO) term enrichment of nucleosome assembly and binding, cytosolic ribosome, and viral RNA replication.

Histones are proteins abundant in the eukaryotic nucleus, also found in extracellular biofluids of healthy individuals and in EV proteomes, but are commonly seen elevated in diseases [38,39]. Upregulation of histone secretion is associated with cellular stress and a shift toward a smaller EV population/exosomes (<200 nm). As the glucose default in commercial media is 11.11 mM, the presence of histones in HBMEC 5.5 mM EVs could indicate cellular stress due to the lower glucose conditions, although the functional role and significance of histones in EVs are still unclear [38]. A concentration of 5.5 mM was defined as a normo-glucose condition because it is equivalent to 99.9 mg/dL, a physiologic fasting plasma glucose. However, we cannot discard the possibility of low-glucose culture stress.

Other distinct proteins enriched or exclusively identified in HBMEC 5.5 mM EVs were fibronectin (FN1), integrin (INTB3), collagens (COL4A1, COL4A5), and tetraspanin CD151. All components of the extracellular matrix (ECM)—which brain endothelial cells, along with pericytes and astrocytes, secrete to constitute the basal lamina—represent an important non-cellular part of the brain–blood barrier (BBB) [40,41]. This profile implies suitable control of endothelial layer permeability under normal glucose conditions. The opposite occurs with a high-glucose EV profile, as these same ECM proteins are expressed at lower levels in HBMEC 33 mM EVs.

In addition to barrier disruption signs, HBMEC 33 mM glucose EVs showed a cargo shift toward metabolism, inflammation, and angiogenesis. The positive regulation of GAPDH and GPI indicates glycolytic activity. The high-glucose condition also increased expression of ANXA2P2, LDHA, and PKM in EVs, which promote the Warburg effect (aerobic glycolysis) [42,43]. Another metabolic protein raised in 33 mM EVs was CD44, involved with insulin resistance in obesity and diabetes [44].

To complete the metabolic profile, fatty acid-binding protein 5 (FABP5) expression reinforces EV signaling toward glycolysis, fatty acid oxidation, and proliferation under high glucose. FABP4/5 aberrant expression was associated with increased glycolysis in lung endothelial cells through reactive oxygen species and the HIF-2α (hypoxia-inducible factor-2α) pathway [45], indicating oxidative stress as well.

GO analysis revealed terms related to endosomal sorting complexes required for transport (ESCRT), multivesicular bodies (MVBs), and syntaxin-1 binding in HBMEC 33 mM EVs. Syntaxin-1 is part of the presynaptic SNARE complex and essential for neurotransmission. The release of neuronal proteins within EVs has been considered a potential biomarker for different pathologies, such as Parkinson’s disease and ischemic stroke [46,47]. Beyond this, alteration in O-linked glycosylation of Syntaxins (1A and 3) was recently described in diabetic retina proteomics [48]. Together with our data, this suggests that hyperglycemia can alter the expression, post-translational modification, and secretion of metabolic and synaptic proteins in endothelial cells.

Other proteins abundantly expressed in the brain, such as BASP1 and RAB11B, were enriched in the HBMEC 33 mM EVs proteome, characterizing diverse alterations in endothelial EV composition under high glucose, despite the decrease in EV release number. Additionally, further signs of barrier dysfunction appeared with proteins related to small GTPase (Rac1/Rhoa) activity, which regulate the cytoskeleton, tight junctions, and adhesion molecule expression [35]. CLIC1, CLIC4, EZR, and IQGAP1 were upregulated in 33 mM HBMEC EVS. CLICs’ function, along with ERM (ezrin/radixin/moesin) phosphorylation, is essential to small GTPase-mediated barrier disruption [49].

Based on these data, the activation of brain endothelial cells by high glucose directly influences the amount of EVs released and their proteomic profile, directing them toward metabolic activity, barrier disruption, and neuronal protein cargo. We hypothesize that high glucose-altered EVs may exacerbate microenvironmental inflammation and enhance monocyte recruitment and cellular infiltration into the tissue.

In this scenario, using our undifferentiated monocyte model, the protein content of THP-1 EVs exhibited inflammatory profiles for both glucose conditions. THP-1 33 mM EVs had positive expression of serine protease cathepsin G (CTSG), indicating high-glucose monocyte activation. Cathepsin G acts directly in antigen presentation, a feature consistent with GO enrichment terms related to immune responses, such as inflammation, cytotoxicity, and MHC-I protein binding.

THP-1 5.5 mM EVs had positive expression of peroxiredoxin 3 (PRDX3), which signals mitochondrial antioxidant function; PRDX3 removes cellular ROS (reactive oxygen species), inhibits apoptosis, and favors cell proliferation [50,51]. This is a common chemoresistance response in cancer, considering that the THP-1 lineage comes from acute monocytic leukemia. The NURF (nucleosome remodeling factor) complex term was highly enriched in our GO analysis of 5.5 mM EVs, which implies cell survival and inflammatory responses in the THP-1 lineage under normal glucose as well.

The activated-inflammatory proteomic EV profile, together with THP-1 EVs’ pro-adhesive effect on the endothelial monolayer, is consistent with descriptions in the literature of high-glucose monocyte/macrophage EV actions, among them the promotion of cellular proliferation and dysregulation of mitochondrial activity [32] and the induction of ICAM-1 expression in endothelial cells [31]. Overall, our data reinforce EVs as mediators of immune cross-talk and show that high-glucose-altered EVs can activate endothelial cells.

Considering our study approach—restricted to in vitro assays using a single endothelial cell line, a cancer-derived monocytic cell—and given the absence of an osmotic control, we have to acknowledge the limitations in generalizing our data to primary cells and/or systemic chronic responses. But based on our observations, high-glucose-altered EVs are an active piece of monocyte/endothelial cell interaction and have potential as inflammatory biomarkers.

Regardless of EVs’ exact protein component and their mechanism of action, our findings also revealed that monocyte-derived EVs may be more pro-adhesive than endothelial-derived EVs. This could explain, in part, the reports of increased circulating leukocytes in diabetic individuals [32]. But this needs to be confirmed with different monocytic and/or primary cell lines.

Further studies labeling and/or blocking the proteins we identified by proteomics, as well as using other cell lineages, can validate the hypotheses we have raised here. An experimental design using osmotic control (such as mannitol or L-glucose) in short- and long-term exposure to elevated glucose levels could distinguish EV release in acute and chronic phases. To date, our observations and proteome descriptions show that exposure to high glucose levels can affect the inflammatory role of EVs; this highlights the importance of population glycemic control, especially in vulnerable groups.

## 4. Materials and Methods

### 4.1. Cell Culture

Immortalized human brain microvascular endothelial cells (HBMEC, Creative Biolabs, Shirley, NY, USA, cat. DBFF-1123-HX2043) and THP-1 undifferentiated monocytes (ATCC TIB-202) were kept in 25 cm^2^ (T25) culture flasks with 5 mL of RPMI-1640 medium, no glucose (Sigma-Aldrich, St. Louis, MO, USA, cat. R1383) added with 5.5 mM (normal glucose) and 33 mM (high glucose) of D-glucose and supplemented with 10% fetal bovine serum (FBS), 2 mM L-glutamine, 50 IU/mL penicillin and 50 µg/mL streptomycin in an incubator at 37 °C and 5% CO_2_.

A batch of frozen HBMECs at passage number 28 was used. When the monolayer reached 90% confluence, the cells were subcultured by adding 1 mL of trypsin (5 mg/mL)-EDTA (2 mg/mL) solution, counted in a Neubauer chamber, and approximately 1 × 10^5^ cells were transferred to new T25 flasks. The medium was changed every three days, or when necessary. To conduct the experiments, a minimum of two and a maximum of six passages were performed in media with different glucose concentrations. For THP-1 subculture, after T25 culture flasks reached approximately 4.3 × 10^6^ cells, the monocytes were centrifuged at 300× *g* for 5 min, then 1 × 10^5^ cells were transferred to new T25 flasks or expanded for the experiments.

### 4.2. Flow Cytometry

First, 1 × 10^5^ HBMEC cells were seeded per well in 6-well plates in medium with either 5.5 mM or 33 mM glucose and incubated at 37 °C with 5% CO_2_. Then, after 48 h, cells were harvested by scraping and stained with Alexa Fluor 488-conjugated Annexin V and propidium iodide (PI) following the manufacturer’s protocol. The gating strategy consisted of untreated and unlabeled cells gated to define the cell population (single cells); cells treated with 10 ng/mL of TNF-α (24 h before) and 2% Saponin (at the moment of labeling) served as positive controls to identify FITC-positive (Annexin V) and PE-positive (PI) populations. A total of 10,000 events of each experimental condition were analyzed by a FACSCanto II flow cytometer (Becton Dickinson, Franklin Lakes, NJ, USA), and data were processed with FlowJo v10.9.0.

For analysis of CD54/ICAM-1 expression, HBMECs cultured with 5.5 mM and 33 mM glucose were seeded in 6-well plates at a density of 2 × 10^5^ cells/well for 24 h. A positive control was included by treating cells with 10 ng/mL of TNF-α. Following treatment, cells were harvested by washing with PBS and incubating with PBS-EDTA (1.5 mM), using a cell scraper. After centrifugation at 300× *g* for 10 min, cell pellets were blocked in PBS-BSA 4% for 20 min. Cells were then sequentially incubated with a primary anti-ICAM antibody (Invitrogen, Waltham, MA, USA, cat. MA1.19028) at a 1:1000 dilution and a secondary anti-mouse IgG-Alexa 488 antibody (Invitrogen, Waltham, MA, USA, cat. A32723) at the same dilution. A total of 10,000 events were analyzed for each experimental condition. Controls included untreated and unlabeled cells, untreated and labeled cells, and PBS-treated cells. The cells were analyzed using a FACSCanto II flow cytometer (Becton Dickinson, Franklin Lakes, NJ, USA), and the data were processed with FlowJo v10.9.0 software.

### 4.3. Immunofluorescence Assays to Detect ICAM-1

First, 3 × 10^4^ HBMEC cells were seeded per well in 24-well plates (on sterile round glass coverslips) in medium with 5.5 mM and 33 mM glucose and were kept in an incubator at 37 °C and 5% CO_2_ until they reached confluency (72 h). Then, the cells were fixed with 4% PFA solution for 5 min, blocked with PBS + 4% BSA for 30 min, and incubated with anti-ICAM-1 (Invitrogen, Waltham, MA, USA, cat. MA1.19028) at 1:200 dilution for 1 h at room temperature. After three 5 min washes with 1× PBS, Alexa Fluor 546 donkey anti-mouse IgG (Invitrogen, Waltham, MA, USA, cat. A10036) secondary antibody (1:600) and 4′,6-diamidino-2-phenylindole (DAPI) (1 µg/mL) were added for 45 min at room temperature. After washing, the coverslips were mounted for viewing under a DMi80 fluorescence microscope (Leica Microsystems, Wetzlar, Germany).

### 4.4. RNA Extraction and RT-qPCR

HBMECs were seeded in T25 culture flasks in media with 5.5 mM and 33 mM glucose. After reaching confluence, they were washed with sterile PBS, and the monolayer was scraped with 1 mL of PBS. The cell samples were immediately kept on dry ice. On the same day, total RNA was extracted using the RNeasy Micro Kit (Qiagen, Venlo, The Netherlands), followed by DNase treatment (Promega, Madison, WI, USA) and reverse transcription to cDNA using a SuperScript™ II Reverse Transcriptase Kit (Invitrogen, Waltham, MA, USA, cat. 18064014), according to the manufacturer’s instructions. Afterward, specific oligonucleotides for VCAM-1 (FW: 5′-AGTTGAAGGATGCGGGAGTA-3′ and RV: 5′-AGAGCACGAGAAGCTCAGGA-3), MCP-1 (FW: 5′-GATCTCAGTGCAGAGGCTCG-3′ and RV: 5′-TTTGCTTGTCCAGGTGGTCC-3′) and IL-8 (FW: 5′-TCTGCAGCTCTGTGTGAAGG-3′ and RV: 5′-ACTTCT CCACAACCCTCTGC-3′) were used for quantitative PCR (qPCR).

The qPCR reaction consisted of the forward and reverse primers at a final concentration of 300 nM each, 5 μL of 2 × SYBR™ Select Master Mix (Applied Biosystems, Foster City, CA, USA, cat. 4472908), 50 ng of cDNA sample, and 18.2 MΩ·cm water to complete a 10 μL reaction. All reactions (four technical replicates per gene) were performed in 96-well plates and analyzed in the QuantStudio™ 5 (Thermo Fisher Scientific, Waltham, MA, USA). The qPCR cycle consisted of stage 1, 95 °C for 10 min (1×); stage 2, 95 °C for 10 s, 60 °C for 30 s (45×), followed by a melt curve: 95 °C for 10 s, 65 °C for 1 min, and 97 °C for 1 s. The oligonucleotides for GAPDH (FW: 5′-GGCCTCCAAGGAGTAAGACC-3′ and RV: 5′-GACTGAGTGTGGCAGGGACT-3′) and β-actin (FW: 5′-AGGATGCAGAAGGAGATCACT-3′ and RV: 5′-GGGTGTAACGCAACTAAGTCATAG-3′) were used as endogenous controls for normalization.

### 4.5. Extracellular Vesicle (EV) Isolation

#### 4.5.1. Preparation of EV-Free FBS

For fetal bovine serum (FBS) EV depletion, FBS pre-inactivated at 56 °C for 30 min was subjected to centrifugation at 100,000× *g* for 18 h at 4 °C in an ultracentrifuge model CP80NX with rotor P32ST (Eppendorf Himac Technologies, Hitachinaka, Japan), then sterilized by filtration through a 0.22 µm filter and stored in 5 mL aliquots at −20 °C until use.

#### 4.5.2. EV Separation and Concentration

For EV isolation from HBMEC, according to [37] cells were cultured in 150 cm^2^ (T150) flasks under complete RPMI-1640 with either 5.5 mM or 33 mM glucose. After the cell monolayer reached approximately 90% confluence, it was washed with PBS, followed by culture medium replacement with 30 mL of the matching RPMI with 10% FBS EV-free, and the cell flasks were placed back in the incubator for 24 h at 37 °C and 5% CO_2_. The next day, EVs were isolated from the cell supernatants by differential ultracentrifugation (dUC) in sequence at 300× *g* for 10 min, 2000× *g* for 10 min, 10,000× *g* for 30 min, and twice at 100,000× *g* for 110 min, all at 4 °C. In the first three centrifugations, the supernatant was collected, then the precipitate formed after ultracentrifugation (containing the EVs) was washed with 10 mL of cold PBS. For the second ultracentrifugation, the P32ST rotor was replaced with a P40ST rotor, and the final EV precipitate was suspended in 50 µL of sterile PBS using a low-intensity vortex and stored in low-binding microtubes at 4 °C for up to one week.

For EV isolation from THP-1, three 75 cm^2^ (T75) flasks per glucose condition were used. Undifferentiated THP-1 cells were cultured in complete RPMI-1640 with either 5.5 mM or 33 mM glucose until reaching approximately 1.3 × 10^7^ cells per flask. Then, THP-1 cells in suspension were harvested and counted again using a Neubauer chamber, washed with PBS (300× *g* for 5 min), and resuspended at 1 × 10^6^ cells/mL in the matching RPMI medium containing 10% FBS EV-free, resulting in 30 mL of culture for each glucose condition. After 24 h at 37 °C and 5% CO_2_, the cultures were centrifuged at 300× *g* for 10 min to remove whole cells (pellet stored), and the supernatant followed the remaining differential centrifugation protocol: 10,000× *g* for 30 min, and 100,000× *g* for 110 min twice, all at 4 °C as described above. The latest centrifugations were performed in the ultracentrifuge model CP80NX with both rotors P32ST and P40ST (Eppendorf Himac Technologies, Hitachinaka, Japan). EVs precipitated from the final centrifugation cycle were suspended in 50 µL of sterile PBS and stored in low-binding microtubes at 4 °C for up to seven days.

Protein quantification for all samples was measured with a Qubit^®^ Protein Assay Kit (Invitrogen, Waltham, MA, USA, cat. Q33211) using a Qubit^TM^ 4 Fluorometer (Thermo Fisher Scientific, Waltham, MA, USA, cat. Q33238) according to the manufacturer’s instructions.

### 4.6. Cell Lysate

Both HBMEC and THP-1 cells used for EV isolation were immediately prepared for protein extraction. After collecting the supernatant from the 24 h HBMEC culture, cells were removed from the flask with a cell scraper, washed with PBS (400× *g* for 10 min), and kept on ice. For THP-1 cells, the whole cell pellet was used. Cells were then suspended in cold PBS with 1% Triton X-100, 1 mM PMSF, and a protease inhibitor cocktail (Roche, Basel, Switzerland) and incubated on ice for 10–15 min. Total protein was quantified using the Qubit^®^ Protein Assay Kit (Invitrogen, Waltham, MA, USA, cat. Q33211) and a QubitTM 4 Fluorometer (Thermo Fisher Scientific, Waltham, MA, USA) according to the manufacturer’s instructions. A 20-µg aliquot of each sample was separated for an SDS-PAGE run and subsequent mass spectrometry analysis.

### 4.7. Nanoparticle Tracking Analysis

Nanoparticle tracking analysis (NTA) was conducted using the Nanosight NS300 instrument from Malvern Instruments, run with NTA software (version 3.4 Dev Build 3.4, Malvern Panalytical, Worcestershire, UK). Samples were appropriately diluted in PBS to achieve approximately 100 particles per frame. Particle tracking employed a 305 nm laser and an sCMOS camera set at level 10, with a detection threshold of 3 and a flow rate of 50. All analyses consisted of five 60 s videos.

### 4.8. Transmission Electron Microscopy

To obtain EV images, glass slides were coated with a thin membrane of 0.5% Formvar in chloroform. Metal grids were placed on the membrane, lifted with paraffin plastic film, and air-dried in a Petri dish for 10 min. Static electricity was neutralized using an anti-static ionizing gun (Zerostat, Coventry, UK). Next, 50 μL of extracellular vesicle samples diluted in PBS (1:10) was applied to the grids, air-dried for 1 h in closed Petri dishes, and excess PBS was carefully removed. Negative contrast staining was performed using 1 mL of uranyl acetate, applied to the grids and left for 20 min in the dark. Following drying, the grids were stored for examination under a JEM-1400Plus transmission electron microscope (JEOL, Tokyo, Japan) at the ICC/Fiocruz-PR Microscopy Platform.

### 4.9. Proteomic Sample Preparation

EV samples were diluted in a buffer with 1% SDS and 50 mM Tris-HCl, pH 8.0. Approximately 10 µg were applied to a 12% SDS-PAGE gel, and electrophoresis was run at 30 mA for 30–40 min, followed by silver nitrate staining (Sigma-Aldrich, St. Louis, MO, USA, cat. S5506). The regions of the gel demarcated with the proteins were cut, separated into microtubes, and completely decolorized. Gel fragments were dehydrated with absolute ethanol and then dried in a SpeedVac. The same procedure was followed with the cell lysates.

Afterwards, the samples were reduced with 10 mM dithiothreitol (DTT) for 60 min at 56 °C and alkylated with 55 mM iodoacetamide (IAA) for 45 min at 25 °C, protected from light. Enzymatic digestion was performed using 50 mM ABC solution with trypsin (12.5 ng/µL) for 20 min at 4 °C. Then, any excess trypsin solution (if present) was discarded. The samples were incubated in new 50 mM ABC solution for 16–18 h at 37 °C. After digestion, the peptides were extracted using a solution of water, 3% TFA, and 30% acetonitrile. Samples were stored at −20 °C until liquid chromatography-mass spectrometry (LC-MS/MS) analysis.

### 4.10. Mass Spectrometry-Based Proteomic Analysis

Peptides were purified with StageTips-C18 and analyzed on an Orbitrap Exploris 120 mass spectrometer (Thermo Fisher Scientific, Waltham, MA, USA) coupled to the UltiMate™ 3000 RSLCnano UHPLC system at the Mass Spectrometry Facility RPT02H, Instituto Carlos Chagas/Fiocruz-PR. Chromatographic separation was performed on a 15 cm analytical column (75 µm i.d., packed with 3 µm C18 particles) at a flow rate of 250 nL/min using a 60 or 90 min linear gradient from 5% to 40% acetonitrile in 0.1% formic acid. The mass spectrometer operated in data-dependent acquisition (DDA) mode with EASY-IC™ internal calibration. DDA isolation window of *m*/*z* 1.6.

Full MS scans (*m*/*z* 300–1500) were acquired at a resolution of 120,000. The top 4 most intense precursor ions were selected for fragmentation via higher-energy collisional dissociation (HCD) with a normalized collision energy of 30%, and MS/MS spectra were acquired at a resolution of 15,000 (first mass: *m*/*z* 200). Dynamic exclusion was enabled for 60 s. The AGC target was set at 100% for MS and standard for MS/MS, with maximum injection times of 50 ms (MS) and 22 ms for MS/MS.

The data processing was performed using MaxQuant, with 81,837 entries from the *Homo sapiens* UniProt database and a label-free quantification strategy. The resulting proteinGroups.txt file was imported into Perseus (version 2.0.11) for statistical analysis. Protein intensities were log2-transformed, and contaminants, reverse entries, and proteins identified only by site were removed. Proteins with missing values in less than 70% of samples in at least one group were excluded. The remaining missing values were imputed based on a normal distribution (width = 0.3, downshift = 1.8). Differential expression analysis was performed using Student’s *t*-test with a false discovery rate (FDR) correction (Benjamini–Hochberg method), considering proteins with *p*-value < 0.05 and log2 fold change ≥ 1 as significantly differentially expressed.

The list of non-shared proteins identified in each group was analyzed for Gene Ontology (GO) using the PANTHER™ Classification System (https://pantherdb.org/) version 19.0. Overrepresentation tests for GO terms of biological process, molecular function, and cellular component were performed using Fisher’s exact test with FDR correction against the *Homo sapiens* reference list.

### 4.11. Cell Adhesion

HBMECs cultured in RPMI medium (Gibco, Waltham, MA, USA, cat. 31800022) with commercial standard 11 mM glucose were seeded at 3 × 10^4^ cells/well in 96-well plates and kept in an incubator at 37 °C and 5% CO_2_ until confluence was reached (48 h). Then, 100 ng/mL per well of isolated EVs from THP-1 under 5.5 mM and 33 mM glucose, or from HBMEC under the same conditions, was added. After 16 h of EV total uptake, 1.2 × 10^5^ cells/well (1:1 ratio) of THP-1 cells labeled with CFSE (Invitrogen, Waltham, MA, USA, cat. C34554) at 5.5 mM and 33 mM glucose, corresponding to each EV treatment, were added. Plates were incubated for 1 h at 37 °C and 5% CO_2_ under slight agitation. Then, the wells were carefully washed four times with PBS and fixed with 4% PFA solution for 5 min. The plates were kept in PBS and analyzed under a DMi80 fluorescence microscope (Leica Microsystems, Wetzlar, Germany) to assess the adhesion of CFSE+ THP-1 monocytes to the HBMEC monolayer. The acquired images were analyzed using ImageJ 1.54g software.

Three independent experiments were performed with three technical replicates for each condition. Controls consisted of untreated wells of RPMI 10% FBS with 5.5 mM and 33 mM glucose (FBS EV: non-human-related EV condition) and wells of RPMI 10% FBS-free medium with 5.5 mM and 33 mM glucose (No EV condition).

### 4.12. Statistical Analysis

Statistical tests were applied based on the characteristics of each dataset. Sample normality was assessed using the Shapiro–Wilk test and/or Kolmogorov–Smirnov (distance) and visually checked using Q-Q plots. Student’s *t*-test was used to compare means between groups with unpaired parametric variables. For multiple comparisons, one-way ANOVA followed by Tukey’s test was used, or multiple *t*-tests (nonparametric) with FDR correction. FDR was determined using the Benjamini–Hochberg procedure, with Q = 1%.

The respective statistical approach is described in the figure legends. Graphs and analyses were generated with GraphPad Prism 8.0.1 (GraphPad Software). Results with *p*-values ≤ 0.05 were considered statistically significant: * for *p* < 0.05, ** for *p* < 0.001 and *** for *p* < 0.0001.

## Data Availability

All data generated or analyzed during this study are included in this published article and its Supplementary Materials.

## Supplementary Materials

The following supporting information can be downloaded at https://www.mdpi.com/article/10.3390/ijms27188107/s1.
